# Molecular Characterization of Three Canine Models of Human Rare Bone Diseases: Caffey, van den Ende-Gupta, and Raine Syndromes

**DOI:** 10.1371/journal.pgen.1006037

**Published:** 2016-05-17

**Authors:** Marjo K. Hytönen, Meharji Arumilli, Anu K. Lappalainen, Marta Owczarek-Lipska, Vidhya Jagannathan, Sruthi Hundi, Elina Salmela, Patrick Venta, Eva Sarkiala, Tarja Jokinen, Daniela Gorgas, Juha Kere, Pekka Nieminen, Cord Drögemüller, Hannes Lohi

**Affiliations:** 1 Department of Veterinary Biosciences, University of Helsinki, Helsinki, Finland; 2 Research Programs Unit, Molecular Neurology, University of Helsinki, Helsinki, Finland; 3 The Folkhälsan Institute of Genetics, Helsinki, Finland; 4 Department of Equine and Small Animal Medicine, University of Helsinki, Helsinki, Finland; 5 Institute of Genetics, Vetsuisse Faculty, University of Bern, Bern, Switzerland; 6 Department of Microbiology and Molecular Genetics, Michigan State University, East Lansing, Michigan, United States of America; 7 Division of Clinical Radiology, Department of Clinical Veterinary Medicine, Vetsuisse Faculty, University of Bern, Bern, Switzerland; 8 Department of Biosciences and Nutrition, Karolinska Institutet, Huddinge, Sweden; 9 Department of Oral and Maxillofacial Diseases, University of Helsinki, Helsinki, Finland; Clemson University, UNITED STATES

## Abstract

One to two percent of all children are born with a developmental disorder requiring pediatric hospital admissions. For many such syndromes, the molecular pathogenesis remains poorly characterized. Parallel developmental disorders in other species could provide complementary models for human rare diseases by uncovering new candidate genes, improving the understanding of the molecular mechanisms and opening possibilities for therapeutic trials. We performed various experiments, e.g. combined genome-wide association and next generation sequencing, to investigate the clinico-pathological features and genetic causes of three developmental syndromes in dogs, including craniomandibular osteopathy (CMO), a previously undescribed skeletal syndrome, and dental hypomineralization, for which we identified pathogenic variants in the canine *SLC37A2* (truncating splicing enhancer variant), *SCARF2* (truncating 2-bp deletion) and *FAM20C* (missense variant) genes, respectively. CMO is a clinical equivalent to an infantile cortical hyperostosis (Caffey disease), for which *SLC37A2* is a new candidate gene. SLC37A2 is a poorly characterized member of a glucose-phosphate transporter family without previous disease associations. It is expressed in many tissues, including cells of the macrophage lineage, e.g. osteoclasts, and suggests a disease mechanism, in which an impaired glucose homeostasis in osteoclasts compromises their function in the developing bone, leading to hyperostosis. Mutations in *SCARF2* and *FAM20C* have been associated with the human van den Ende-Gupta and Raine syndromes that include numerous features similar to the affected dogs. Given the growing interest in the molecular characterization and treatment of human rare diseases, our study presents three novel physiologically relevant models for further research and therapy approaches, while providing the molecular identity for the canine conditions.

## Author Summary

Rare developmental disorders make a major contribution to pediatric hospital admissions and mortality. There is a growing interest in the development of therapeutics for these conditions, but that requires understanding of the genetic cause and pathology. Research can be facilitated by physiologically relevant models, such as dogs with corresponding disorders. We have characterized the clinical features and genetic causes of three developmental syndromes in dogs, including craniomandibular osteopathy (CMO), a previously undescribed skeletal syndrome, and dental hypomineralization, for which we identified mutations in the canine *SLC37A2*, *SCARF2* and *FAM20C* genes, respectively. CMO is a clinical equivalent to an infantile cortical hyperostosis (Caffey disease) for which *SLC37A2* is a new candidate gene. SLC37A2 is a glucose-phosphate transporter in osteoclasts, and its defect suggests an impaired glucose homeostasis in developing bone, leading to hyperostosis. Mutations in the *SCARF2* and *FAM20C* genes have been associated with the human van den Ende-Gupta and Raine syndromes. Our study provides molecular identity for the canine conditions and presents three novel physiologically relevant models of human rare diseases.

## Introduction

One to two percent of all children are born with a developmental disorder, such as a heart defect, skeletal abnormality, or mental retardation as a result of errors in embryogenesis and early neurodevelopment. These disorders make a major contribution to pediatric hospital admissions and mortality [1]. Rare pediatric disorders are typically with homozygous, compound heterozygous, or *de novo* pathogenic variants. The advent of cost-efficient next generation sequencing (NGS) technologies drives gene discovery in many disorders without a cognate gene [2] and thousands of rare variants have been described (www.orpha.net). There is also a growing interest in the development of therapeutics for rare diseases, which requires the identification of the genetic defects, comprehensive understanding of the molecular pathology and access to physiologically relevant animal models.

Developmental disorders are frequent also in other species, including dogs, which as large animals bear very close physiologic and genetic resemblance with us. Dogs give birth to litters with multiple puppies. However, often some of the littermates are affected by developmental or other abnormalities, and perinatal mortality (stillbirth, fetal and neonatal death) is common with prevalence ranging from 5 to 35% [3]. The causes include respiratory distress syndrome/hypoxia, infectious diseases, severe malformations and suspected hereditary diseases. Culling is a common practice among dog breeders and the deceased or abnormal puppies are not always presented to the veterinarian. Therefore, numerous syndromes with likely genetic origin remain unknown. It would be important to investigate the extent of this common phenomenon at the clinical and molecular level to better understand the diverse causes of the morbidity and to better manage it through advised breeding programs. At the same time, the identification of the causative gene could inform gene functions, disease etiology, molecular pathology and phenotypic overlap across species. Importantly, physiological similarity of dogs with human would establish relevant therapeutic models to human rare disorders. Online Mendelian Inheritance in Animals (OMIA), a catalogue of inherited disorders and associated genes in animals, reports more than 350 inherited diseases in dogs as potential models for human disease (http://omia.angis.org.au/).

NGS approaches are rapidly changing the diagnostic landscape in veterinary medicine in companion animals and enable now a feasible approach to tackle the molecular background of developmental conditions in small pedigrees with translational potential to human rare disease. For example, we have previously discovered a new gene (*ATG4D*) responsible for a neurodegenerative vacuolar storage disease in Lagotto Romagnolos [4] and a missense variant in canine *FAM83G* causing palmoplantar hyperkeratosis and demonstrating its role in maintaining the integrity of the palmoplantar epidermis [5]. A *CNGB1* frameshift variant has been identified to cause a progressive retinal atrophy in dogs [6] and the same gene has been associated with retinal degeneration in human as well [7]. Similarly, we have found that a congenital skeletal disease in Brazilian Terriers is caused by a pathogenic variant in the *GUSB* orthologue responsible for human pediatric disorder, mucopolysaccharidosis type VII [8].

This study addressed the clinical and genetic background of three developmental disorders in dogs; craniomandibular osteopathy (CMO; OMIA: 000236–9615) in West Highland White Terriers (WHWT), Cairn Terriers and Scottish Terriers, a previously undescribed developmental syndrome in Wire Fox Terriers, and dental hypomineralization in Border Collies. CMO is a self-limiting proliferative bone disease seen in young dogs [9]. It manifests between 4 to 8 months of age with typical signs including swelling of the jaw, periodical fever, lack of appetite, pain, difficulty opening the mouth and dysphagia. The excessive proliferation causes bony lesions primarily on the skull bones, especially on the mandible and tympanic bulla, but occasionally also on the metaphyses of long bones. Signs of the disease usually resolve with time, when the growth period is finished. CMO exists in several breeds with the highest frequency in WHWT and has been suggested to be an autosomal recessive trait [10–12]. Canine CMO corresponds to human Caffey disease [MIM: 114000] and its genetic characterization might reveal insights into similar painful human swelling disorders [10]. We identified a novel CMO gene that represents a candidate gene for human Caffey disease. The two other syndromes have not previously been reported in dogs. We describe the detailed clinical features for them in our study. Moreover, we demonstrate their shared genetic etiology with the corresponding human syndromes, van den Ende-Gupta (VDEGS [MIM: 600920]) and Raine syndromes [MIM: 259775].

## Results

### A splicing variant in SLC37A2 causes craniomandibular osteopathy in three terrier breeds

**Fig 1:**
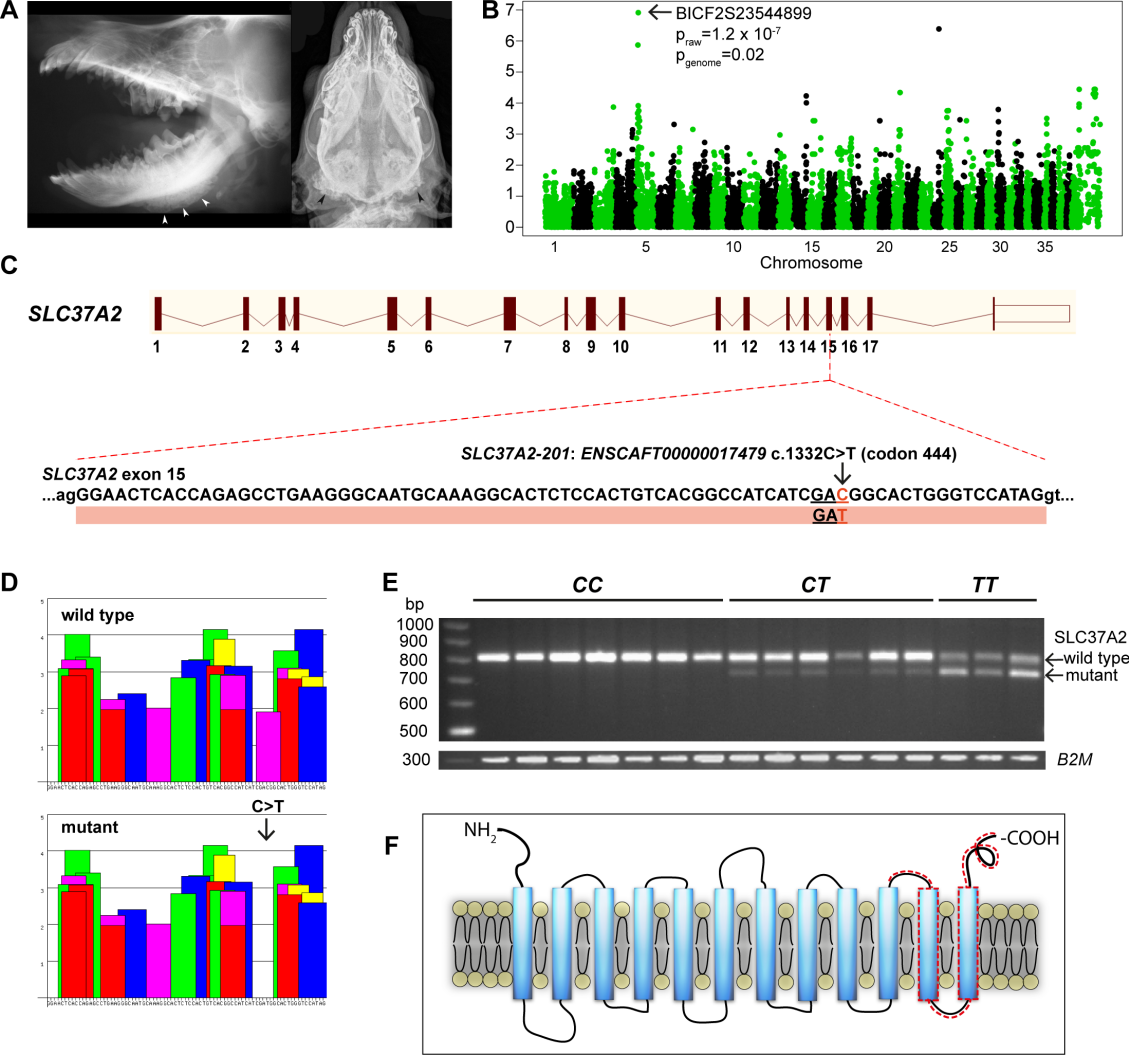
Genetic analyses in canine craniomandibular osteopathy identify a leaky splicing variant in exon 15 of *SLC37A2*, resulting in a frameshift that truncates the protein. **A**. Laterolateral (left) and ventrodorsal (right) radiograph of the skull of two different WHWT affected with CMO. The typical palisade-like periosteal new bone formation is commonly located along the corpus mandibulae (left, white arrowheads) or around the Bulla tympanica (right, black arrowheads). **B.** Manhattan plot from GWAS indicates the associated region on chromosome 5 (p_genome_ = 0.02). **C.** A synonymous variant, c. 1332C>T, in exon 15 of *SLC37A2*. **D**. ESEfinder suggests that the synonymous variant affects a splicing enhancer site. The binding scores for the different splicing factors (colored blocks) for 79 nucleotides of exon 15 are shown for the wild-type and mutant allele. The c.1332C>T mutation eliminates a potential binding site for the SF2/ASF splicing factor (shown in purple). Binding scores for different splicing proteins (shown on the y-axis) are based on different weight matrices and cannot be compared directly to each other. **E**. A semi-quantitative RT-PCR in the wild-type (CC), carrier (CT) and affected (TT) WHWTs confirms the splicing defect (79 bp deletion) caused by the variant, and reveals a splicing leakage in the carrier and affected dogs. *B2M* was used as a loading control. **F**. A schematic 12-transmembrane secondary structure of SLC37A2 predicted by TMHMM. The truncated region in the mutated protein is indicated by a dashed line.

We performed a genome-wide association study (GWAS) to map the CMO locus with Illumina’s 22K canine SNP chips in a cohort of 51 WHWTs, including 10 cases (diagnosed by radiography; **Fig 1A**) and 41 controls. A case-control association test revealed significant association on CFA5 with the best SNP (BICF2S23544899) at 8,953,507 Mb (p_raw_ = 1.2 x 10^−7^, pgenome = 0.02) (genomic inflation factor λ = 1.17) (**Fig 1B**). Manual assessment of genotypes at the CFA5 locus revealed a shared 1.9-Mb homozygosity block in all affected dogs spanning from 7,764,955 bp to 9,707,794 bp. The same homozygosity block was seen in five controls as well. The associated region was replicated and fine-mapped using 105 additional SNPs in 88 samples from three related breeds, WHWT, Cairn Terriers and Scottish Terriers. Fine mapping confirmed the association in all three breeds with the best SNP BICF2S23134295 at 8,183,669 (p = 2.09x10^-15^).

To identify candidate variants, the associated and fine mapped region (1.8 Mb) was captured from two affected and two healthy WHWT with opposite haplotypes followed by a paired-end NGS by HiSeq2000. The shared variants identified in the two affected WHWT dogs were filtered against the two WHWT controls and 32 additional healthy dogs from four different breeds. We found altogether three homozygous variants shared in cases **(S1 Table);** two intergenic indels and a synonymous variant in exon 15 of solute carrier family 37 member 2 gene (*SLC37A2* c.1332 C>T) **(Fig 1C**). As an additional independent verification to avoid potential targeted capture biases, we performed whole genome sequencing in one CMO-affected WHWT and 188 control dogs from other breeds (**S2 Table**) to compare the variants in the associating region. This analysis yielded a single case-specific variant (chr5 g.9,387,327G>A) that was the same as the one identified in the capture experiment.

Although the variant, c.1332C>T in exon 15 of *SLC37A2* is synonymous, it was predicted to affect a splicing enhancer element based on ESEfinder analysis (**Fig 1D**). The mutant *T* allele eliminates a potential binding site for the splicing factor ASF/SF-2. To confirm the predicted effect on splicing, we amplified the region between exons 7 and 18 in lymphocyte mRNAs from three cases, six carriers and seven controls. RT-PCR experiments showed that two alternatively spliced *SLC37A2* transcripts were expressed in all dogs that carried one or two copies of the mutant *T* allele at the *SLC37A2* SNP, regardless of disease status (**Fig 1E**). Sequencing of the RT-PCR products indicated that the smaller band corresponded to a mutant *SLC37A2* transcript lacking 79 bp from exon 15. The splice variant resulted in a frameshift and premature stop codon at the beginning of exon 16. This altered splicing was predicted to lead to a C-terminally truncated protein lacking 75 amino acids compared to the wild-type SLC37A2 protein (**Fig 1F**). The RT-PCR indicated that both wild-type and mutant transcripts were expressed in even the CMO affected dogs, although the expression of the wild-type transcript was significantly reduced in the affected homozygous dogs (**Fig 1E**). The reduction of the wild type transcript level was more moderate in healthy heterozygous carrier dogs. None of the examined wild-type dogs expressed the mutant transcript. Overall, these results demonstrate the leaky nature of the splice site mutation.

To investigate the segregation and frequency of the variant across the CMO affected breeds, we performed a large variant screening by genotyping the c.1332C>T variant altogether in 1052 dogs, including 695 WHWT, 249 Scottish Terriers and 108 Cairn Terriers (**S3 Table**). We found 123 homozygous dogs in the WHWT breed, of which 48% had been reported with CMO. About 40% of WHWT (275 dogs) carried the pathogenic variant, of which 10 dogs (3,6%) were reported with CMO. In Scottish Terriers, 10 dogs (4%) were homozygous and all were reported with CMO, and 43 dogs (17%) were carriers, of which 3 dogs (7%) were reported with CMO. In Cairn Terriers, 9 dogs (8%) were homozygous and reported with CMO, and 15 dogs (14%) carried the pathogenic variant, from which 3 dogs (20%) were reported with CMO. We found one wild-type dog in both Scottish and Cairn Terriers with CMO, and screened the coding regions of the entire *SLC37A2* gene in these two dogs for possible other pathogenic variants, but did not find any. This suggests phenocopies, misdiagnoses or genetic heterogeneity. The analysis of the pathogenic variant in the three main breeds with 96 cases resulted in a highly significant association (p = 6.62x10^-303^) with CMO.

In addition to the above three breeds, we screened the c.1332C>T variant in 458 dogs in 124 breeds, but found only a single heterozygous carrier dog in Jack Russell Terrier breed (**S3 Table**). The phenotype information for this dog was not available. The variant was also screened in the known CMO cases from seven breeds (two Bull Terriers, one Curly Coated Retriever, two Border Collies, one Australian Terrier, one Basset, one German Wirehaired Pointer, one Old English Sheepdog), but they did not have it.

Collectively, our results suggest that CMO is inherited as dominant disease with incomplete penetrance. Canine CMO is equivalent to Caffey disease and our data reveals a novel candidate gene, *SLC37A2*, for the syndrome.

### Truncated SCARF2 causes a skeletal syndrome in Wire Fox Terriers

Wire Fox Terrier breeders contacted us for help in the characterization of an unknown congenital syndrome with severe mandibular prognathia and other skeletal features, mainly severe patellar luxation, in the breed. Two affected 7-week-old puppies from different litters, two unaffected littermates and two affected adult Wire Fox Terriers were examined by radiography. Additionally, a computed tomography (CT) study of the skull was made on two affected dogs (one adult and one puppy), and three dogs were studied for general clinical characteristics and neurological examination.

**Fig 2:**
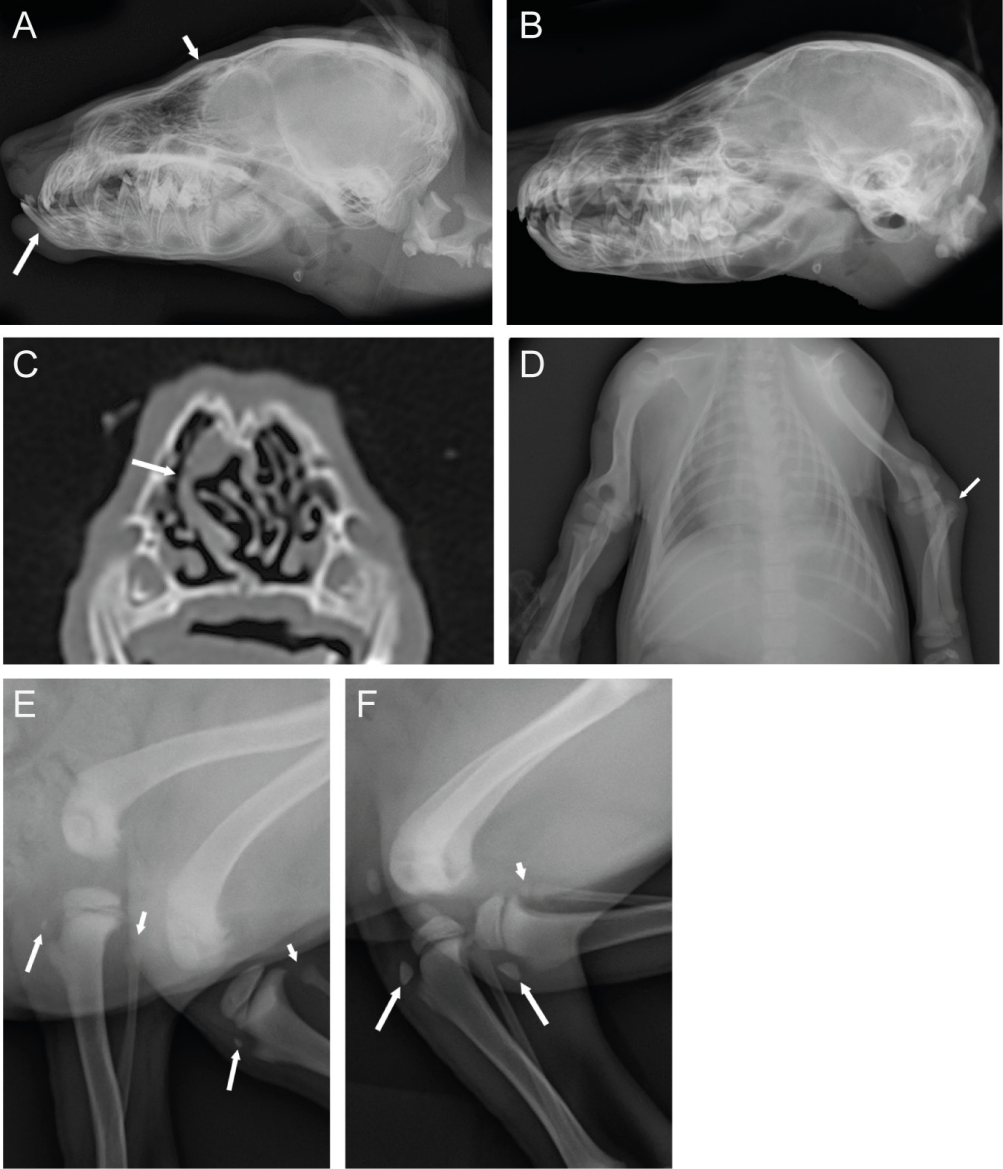
Radiographic and CT findings in a novel canine developmental syndrome. **A**. Lateral radiograph of a 7-week-old affected Wire Fox Terrier puppy, where prominent underbite (long arrow) and dorsally convex maxilla (short arrow) are clearly visible. **B**. Healthy littermate with normal scissor bite and flat dorsal border of the maxilla. **C**. Transverse CT image of the nasal cavities of an affected puppy. Marked lateral deviation of the nasal septum is evident (arrow). **D**. Lateral radiograph of stifle joints of an affected puppy. The ossification centers are poorly mineralized (long arrows) and the proximal fibular epiphyses are non-mineralized (short arrows). **E**. Lateral image of an unaffected puppy indicates that the ossification centers are normal for the age (long arrows) and proximal epiphysis of the fibula is clearly seen (short arrow). **F**. Ventrodorsal radiograph of an affected puppy. Lateral luxation of the left radial head is evident.

A prominent underbite with short maxilla (brachygnathia superior) was evident in all affected Wire Fox Terriers except one adult dog (**Fig 2A and 2B**). The caudodorsal border of the maxilla was slightly convex in all affected animals. In CT images, the nasal septum deviated prominently to the left at the level of the dorsocaudal frontal bone in both examined dogs (**Fig 2C**). The number and position of the vertebrae were normal, but the mid-thoracic spinous processes were thinner, longer and more horizontally aligned in the affected than in the normal dogs. One adult dog had an abnormally wide second rib. The adult dog had marked spondylosis of the spine. One affected puppy had unilateral congenital elbow luxation (**Fig 2D)**, and in the other the secondary ossification centers of the olecranon were non-mineralized. The secondary ossification centers of the tibial tuberosities were small in both affected puppies, when compared to a healthy puppy (**Fig 2E and 2F**). The proximal epiphyses of the fibulae were not mineralized in the affected puppies and the patellae were medially luxated. The femurs of the affected dogs had medial bowing of mid-shafts of the bone. In an eye examination of a puppy and two affected adult dogs, the eyes appeared small and the sclera thinner than normal.

Clinical examination of three affected dogs indicated swollen knee joints and patellar luxation. Neurologically, all the examined dogs were alert and exhibited no remarkable neurological deficits. Postmortem examination of two puppies (one newborn and one 7 weeks old) did not reveal any additional gross abnormalities.

**Fig 3:**
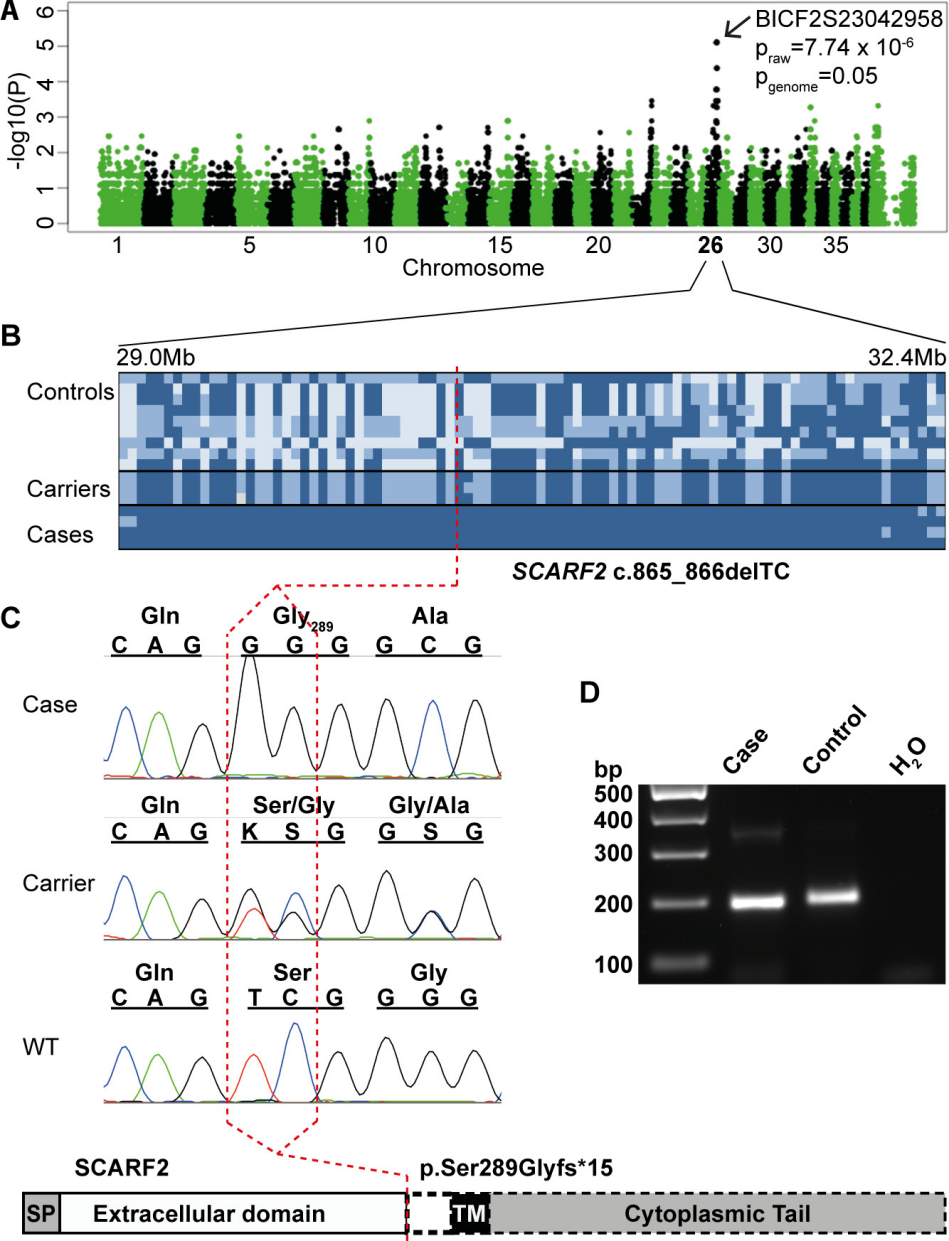
Genetic analyses in a developmental syndrome in Wire Fox Terriers reveal a deletion in exon 6 of *SCARF2*, resulting in an early truncation of the predicted protein. **A**. A Manhattan plot from a GWAS indicates a locus on chromosome 26 (p_genome_ = 0.05). **B**. A 3.3-Mb disease-associated haplotype in the affected dogs. **C**. Targeted resequencing revealed a 2-bp deletion in exon 6 (c.865-866delTC), resulting in a severe truncation at Ser289 that removes the transmembrane and cytoplasmic domains of the predicted protein as indicated by a dashed line in the schematic structure. **D**. RT-PCR in the affected and control Wire Fox Terrier indicate expression of *SCARF2* mRNA and suggest that the mutated *SCARF2* transcript is not directed to nonsense-mediated RNA decay.

To identify the genetic cause of the syndrome, we performed GWAS with Illumina’s CanineHD array in a cohort of 12 Wire Fox Terriers including 4 cases and 8 controls. A case-control association test revealed association on chromosome 26 with seven nearby SNPs at 29,607,333 to 31,863,083 Mb (p_raw_ = 7.74x10^-6^, pgenome = 0.05) (genomic inflation factor λ = 1.10) (**Fig 3A**). Manual assessment of genotypes at the CFA5 locus revealed a shared homozygosity segment of ~3 Mb in the affected dogs spanning from 29,176,909 to 32,226,403 bp (**Fig 3B**). The associated region was captured and resequenced from five samples including two affected, two healthy and one obligate carrier Wire Fox Terrier (**S4 Table**). We identified a 2-bp homozygous deletion in exon 6 of the *SCARF2* gene in the affected dogs after filtering the data according to an autosomal recessive model and against additional 169 unaffected control dogs from different breeds (**S2 Table**). The identified *SCARF2* c.865_866delTC variant results in a frameshift and a premature stop codon, (p.S289Gfs*15), leading to a truncated protein in the first half of the coding region (**Fig 3C**).

Screening of the variant within the Wire Fox Terrier breed (57 dogs) confirmed full segregation with the disease (**S5 Table**). The four cases from the GWA study and an additional case were homozygous for the variant, while obligate carriers were heterozygous. The larger screening for the mutation revealed one homozygous dog that was a littermate of one of the genotyped affected dogs. This dog was then clinically examined, including neurological examination, radiography and CT scanning. The clinical findings confirmed the disease, including mandibular prognathia and patellar luxation. The carrier frequency among the population controls (n = 45) was 22%.

The effect of the 2-bp deletion on the stability of the *SCARF2* mRNA was investigated by RT-PCR in postmortem skin samples from one affected and one unaffected dog (**Fig 3D**). The *SCARF2* mRNA was detected both in affected and unaffected samples suggesting that the mutated transcript is not affected by nonsense-mediated RNA decay in the studied tissue.

*SCARF2* defects have been reported in the rare human bone disease van den Ende-Gupta syndrome. Our results thus established an orthologous canine model with clinical similarity.

### A missense variant in FAM20C causes dental hypomineralization in Border Collies

We were approached by a Border Collie breeder with a family of several affected dogs that suffered from severe tooth wear resulting in pulpitis and requiring extraction of those teeth. Further inspection of the tooth problem in the breed identified additional related cases, suggesting an autosomal recessive mode of inheritance (**S1 Fig**). Two affected dogs were subjected to a clinical study, including dental examination and radiography, as well as to histology of the extracted teeth and were regularly followed up in the next years. In addition, dental radiographs were available from two other cases.

**Fig 4:**
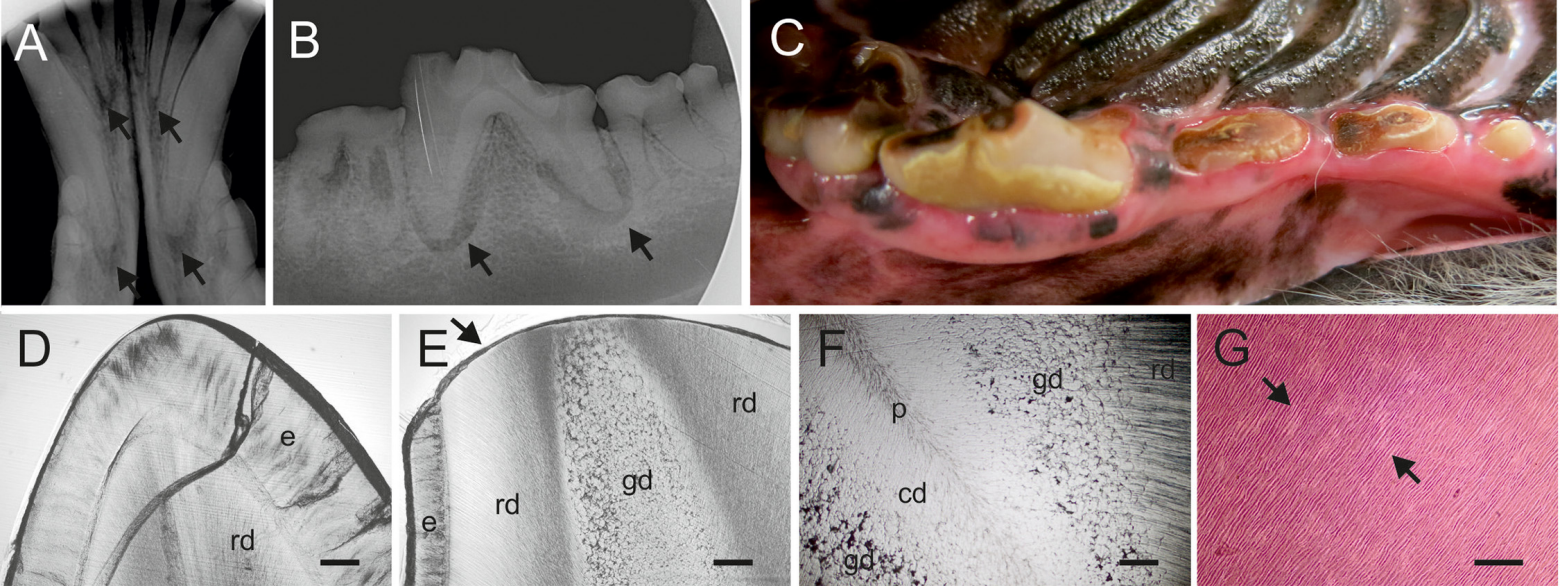
The dental phenotype in the Border Collies with a homozygous *FAM20C* variant. **A.** X-ray of the anterior mandibular teeth of a 9-year-old female Border Collie. Arrows indicate apical inflammations. **B.** X-ray of the anterior mandibular molars and premolars of a 10 year old male Border Collie indicating dental wear and apical inflammations (arrows). **C.** Anterior mandibular molars and premolars of the same dog indicating dental wear causing pulpal exposure. **D.** Ground section of a deciduous incisor from an unaffected Border Collie showing intact enamel and normal dentin, the crack is artefactual. **E.** Ground section of a permanent incisor of an affected 9-year-old female Border Collie. The enamel layer (e) preserved cervically is thin as compared to the control tooth (D). Wear at the edge (arrow) of tooth extends to the peripheral regular dentin (rd) and deeper to the abnormal globular dentin (gd), not present in the control (D). **F.** Ground section from deeper position of the same tooth as in (E) showing the layerwise alteration in the dentin structure. Peripheral dentin (rd) has a normal tubular pattern with no globules, the middlemost zone is pronouncedly globular (gd), while the central zone (cd) next to the diminished or almost completely obliterated pulp chamber (p) shows a slightly irregular tubular pattern but no globules. **G.** Haematoxylin-eosin stained paraffin section of a premolar from a 9-year old female. Contiguous interglobular spaces (arrows) in the middlemost dentin zone stain lightly with eosin. Tubules run uninterruptedly in stained areas. Bars, 50 um (D,E,F) and 200 um (G).

Dental examination of a neutered 9-year-old female Border Collie revealed that all remaining teeth had significant wear. The previous dental treatment was performed 2 years earlier and multiple teeth were extracted. The length of the crowns was reduced. Lower incisor teeth were worn close to gingival margin. The enamel appeared dull and had light brown discoloration. The worn occlusal surfaces were discolored dark brown and there was reparative dentin formation. There were five teeth that had pulp exposure and pulpitis as a result of the wear (**Fig 4A**). The dog’s occlusion was normal and, therefore, the dental wear was not caused by abnormal tooth-to-tooth contact (attrition). Calcitriol (1,25(OH)2D3), phosphate and alkaline phosphatase levels in blood were normal. The other dental examination was performed for a neutered 10-year-old male Border Collie, revealing similar findings (**Fig 4B and 4C**). Other external causes such as abrasive hard chews were excluded as a cause of dental wear in both affected dogs.

Extracted teeth from two affected dogs, a 9-year-old female Border Collie described above and its female littermate, were submitted to histopathological examinations. The analysis of ground sections did not reveal structural aberrations but the enamel of the incisor was smooth and slightly hypoplastic as compared to the unaffected control dog (**Fig 4D and 4E**). The enamel of the premolar had largely worn and cracked, but the cervical enamel, which was preserved, showed no structural defects. Coronal dentin of both teeth comprised three distinct, circumferential zones. The tubular pattern and the structure of the matrix in the thick peripheral zone subjacent to the enamel were regular. The middlemost zone was pronouncedly globular (**Fig 4E and 4F**). The neighboring globules largely adapted to each other and the tubules ran uninterrupted. Wider interglobular spaces were filled with air. The globules diminished and disappeared in the central direction. The central dentin zone next to the reduced pulp showed no matrix defects, but the tubular pattern was slightly irregular (**Fig 4F**). The proportion of globular dentin gradually reduced in the apical direction.

The analysis of paraffin sections demonstrated significant wear of the dentin. The structure of the peripheral zone was regular, whereas the middlemost zone was globular. Between the globules there were wide, contiguous defects with an angular contour, void of matrix and tubules and filled with amorphous, barely detectable material (**Fig 4G**). A pulp chamber did not become visible at the level of the sections. Tubules in the central dentin zone were slightly irregular. Pulp tissue in the root canal was necrotic. A patchy, chronic inflammatory cell infiltrate with plasma cells predominating was present apically. The structure of the acellular cementum was regular. Lacunae in the cellular cementum and at the periphery of the alveolar bone trabeculae were in places obliterated. Demarcated areas in the periodontal ligament facing the alveolar bone showed an amorphous, fibrotic texture. Overall, clinical and histopathological analyses indicate severe hypomineralization of teeth in the affected dogs.

**Fig 5:**
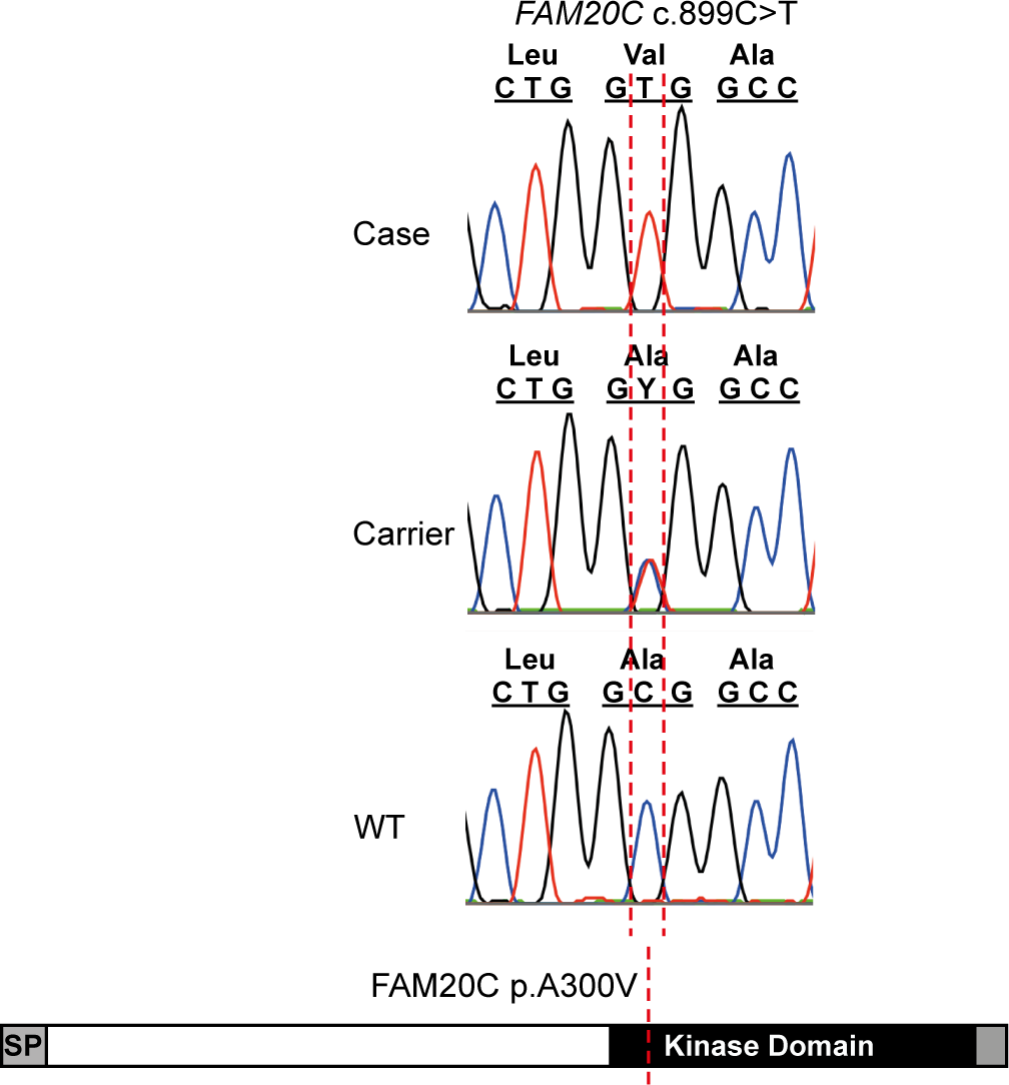
Whole genome sequencing of three Border Collies affected with tooth attrition reveals a missense variant, *FAM20C* c.899C>T, in the conserved kinase domain of the protein (p.A300V).

To identify the cause of the mineralization defect we sequenced the whole genomes of three affected dogs. The variants of the affected dogs were filtered against two unaffected obligate carriers and fourteen other unaffected Border Collie genomes assuming recessive transmission, resulting in the identification of a case-specific non-synonymous homozygous variant, c.899C>T, in the *FAM20C* gene. This leads to a missense change, p.A300V, in a highly conserved position in the kinase domain of the FAM20C protein (**Fig 5**). Bioinformatic predictions by SIFT (with a score of 0.00) and Polyphen2 (with a HumVar score of 0.992) suggested pathogenicity. Genotyping the pedigree and additional Border Collies (191 dogs) demonstrated complete segregation of the variant with the disease phenotype (**S1 Fig and S6 Table**) and showed 11% carrier frequency in the breed. We also genotyped 186 dogs from 20 additional breeds (**S6 Table**), but did not find any carriers in the other breeds suggesting that this pathogenic variant is specific for Border Collies.

Defects in *FAM20C* have been associated with a rare human disease, Raine syndrome/FGF23-related hypophosphatemia characterized by dental and bone hypomineralization. Our results indicated a causative variant in the kinase domain of FAM20C and established a canine model for human Raine syndrome.

## Discussion

This study demonstrates the power of NGS approaches to establish molecular diagnosis and categorization for unknown congenital canine disorders with high relevance to rare human diseases. We identified a novel candidate gene, *SLC37A2*, for the corresponding human disease, infantile cortical hyperostosis, also known as Caffey disease, and implicated *SCARF2* and *FAM20C* variants in the canine forms of van den Ende-Gupta and Raine syndromes, respectively. All three spontaneous canine models closely resemble the human syndromes and provide physiologically relevant models to better understand poorly characterized gene functions in each condition and the entire molecular pathologies. Given the growing interest in the development of new therapies for rare human diseases, our study highlights the fact that dogs carry clinically and genetically close counterparts of rare human diseases that could be better utilized to advance molecular research and to develop efficient preclinical trials in large animals with spontaneous diseases [13, 14]. Many human rare disease models exist in dogs, and encouraging private pet owners to participate in clinical trials could facilitate the validation of human treatment approaches, while also benefitting canine health.

### Identification of the canine CMO variant implicates a sugar-phosphate transporter in hyperostosis

Our study unraveled a physiological function of SLC37A2 and provided new insights into infantile swelling diseases, which may be related to disturbances in the intracellular glucose homeostasis during bone development. We identified an unusual leaky splicing defect in the *SLC37A2* gene in CMO-affected dogs. CMO is a self-limiting hyperostosis in multiple bones in young dogs. The most prominent sign in the affected puppies included painful swelling of the jaw, leading to dysphagia and difficulty in opening the mouth. CMO is clinically equivalent to human infantile cortical hyperostosis [10, 15]. A common missense variant in *COL1A1* has been found in several patients with an autosomal dominant condition with incomplete penetrance [16, 17]. Interestingly, the mechanism how the defected collagen leads to self-limiting hyperostotic bone lesions is still unknown. Early molecular diagnosis of Caffey patients would avoid invasive procedures, however, the molecular etiology remains unknown in many cases.

*SLC37A2* represents a new functional candidate gene. It belongs to the SLC37 family of four ER-associated glucose-phosphate transporters [18]. SLC37A2 is ubiquitously expressed, but transcript and protein levels are particularly high in bone-related tissues such as bone marrow and hematopoietic cell linages such as osteoclasts and macrophages [19, 20]. Murine *Slc37a2* was shown to be one of the genes strongly involved in the osteoclast differentiation, suggesting that it plays a role in osteoclast function and differentiation [20]. Therefore, SLC37A2 may play a central role in glucose homeostasis in the key cell types that participate in osteogenesis. For example, an impaired function of SLC37A2 due to a truncating splice variant might disturb proper glucose supply in the osteoclasts, decreasing their overall activity, which in turn would result in an imbalance between osteoblastic and osteoclastic functions in the developing bones eventually leading to hyperostosis. Defects in *SLC37A4*, glucose-6-phosphate transporter (G6PT), have been associated with glycogen storage disease 1b and 1c, characterized by recurrent infections and neutropenia due to disturbed blood glucose metabolism [21–23]. Our results link SLC37A2 to bone physiology and disease, and we propose *SLC37A2* as an excellent candidate for genetic screening in Caffey patients. Meanwhile, the affected dogs provide unique resources for future experiments to address SLC37A2-related mechanisms in osteogenesis biology. A recent study in hematopoietic cells identified *SLC37A2* as a primary vitamin D target with a conserved vitamin D receptor-binding site [24]. This may open investigations to study the opportunity to use vitamin D as a therapeutic booster to regulate diminished expression of wild type expression of *SLC37A2* in the affected dogs to alleviate clinical signs.

Caffey disease is an autosomal dominant disease with incomplete penetrance, although rare cases of recessively inherited Caffey disease have also been reported [25]. Corresponding canine diseases exist in several terrier breeds with the highest frequency in West Highland White Terriers. The determination of the exact mode of inheritance in dogs is not straightforward due to the nature of the leaky splice variant and mild self-limiting phenotype that may remain unobserved and prevent retrospective diagnosis. We found some dogs that were homozygous for the variant but had no reported clinical signs. However, we observed a considerable level of the wild-type *SLC37A2* transcript in homozygous dogs in the peripheral blood due to the splicing leakage, suggesting that the leaky expression is sufficient to avoid a clinical phenotype in some cases. We also found several heterozygous dogs that had developed CMO. We found that heterozygous dogs had lower levels of wild-type *SLC37A2* transcript compared to the unaffected dogs with individual variation of expression between dogs. This result suggested a dominant disease with incomplete penetrance that could help to explain the reported differences in the severity and duration of CMO among the affected dogs, although alternative models of inheritance cannot be completely ruled out yet. The dominant phenotype could be due to a dominant-negative effect, but this hypothesis requires further experimental validation to better understand the details of the gene, its regulation and protein function, including potential pairing with other proteins as described for SLC37A4/G6Pase complexes [18]. The *in vivo* function of SLC37A4 has been shown to depend upon its ability to couple functionally with either G6Pase-a or G6Pase-b [18, 26].

### A SCARF2 truncation in a canine model of van den Ende-Gupta syndrome

We identified a 2-bp deletion in *SCARF2* in dogs with severe mandibular prognathia and other skeletal abnormalities and established a canine model for van den Ende-Gupta syndrome (VDEGS). VDEGS is a very rare disease with less than 30 reported cases [27]. It is characterized by a heterogeneous variety of craniofacial and skeletal abnormalities including blepharophimosis, a flat and wide nasal bridge, narrow and beaked nose, hypoplastic maxilla with or without cleft palate and everted lower lip, prominent deformed ears, down-slanting eyes, arachnodactyly, and camptodactyly. Patients may present congenital joint contractures that improve without intervention, and have normal growth and development. Enlarged cerebellum is an infrequent finding yet intelligence is normal. Some patients experience respiratory problems due to laryngeal abnormalities. Human and canine VDEGS patients share many similarities including hypoplastic maxilla, dislocated radial head, patellar dislocation, and deviated nasal septum. Both have small eyes.

It remains unknown how the loss of function of SCARF2 leads to VDEGS. SCARF2 is a poorly characterized member of the scavenger receptor type F family [28]. Besides epidermis, *Scarf2* is expressed in branchial arches, mandible, maxilla and urogenital ridge tissue of developing mouse embryos [29, 30]. It is a single-pass transmembrane protein with homology to calmodulin (CaM)-like Ca^2+^-binding protein genes. The extracellular domain contains several putative epidermal growth factor-like (EGF) domains, and it has a number of positively charged residues within the intracellular domain, suggesting a role in intracellular signaling. The 2-bp deletion of the canine *SCARF2* gene in one of the extracellular EGF domains leads to a severely truncated protein that completely lacks the transmembrane and intracellular domains. The lack of a transgenic mouse model and scarcity of human patients highlight the role of affected dogs as a novel resource to understand SCARF2 functions and molecular pathology. As some of the affected dogs survive past 10 years, they could potentially serve also as preclinical models.

### A missense variant in FAM20C identifies a canine model of Raine syndrome

Whole genome sequencing of a family of several affected dogs that suffered from a severe dental wear and loss of teeth revealed a recessive missense variant in the kinase domain of the *FAM20C* gene. *FAM20C* defects cause autosomal recessive osteosclerotic bone dysplasia (Raine syndrome) in humans. This rare syndrome with less than 40 reported cases was originally described to be neonatal lethal, but recently there have been several reports of cases surviving into childhood with variable severity and clinical heterogeneity [31–38]. Typical characteristics in Raine syndrome include craniofacial anomalies, such as exophthalmos, abnormal and hypomineralized teeth, midface hypoplasia, microcephaly and cleft palate, as well as gingival hyperplasia, generalized osteosclerosis and intracerebral calcifications. Variable extent of hypophosphatemia has been observed, sometimes as the primary diagnosis [32, 35, 38]. Canine findings in our cohorts were more limited to severe hypomineralization of teeth, leading to extensive wear and inflammation as prominent features. We did not observe some of the typical gross changes described in Raine patients such as hypophosphatemia and craniofacial anomalies. However, there is a significant clinical heterogeneity in the symptoms between human patients and more detailed radiographic analyses should be performed in dogs to observe potential mild changes outside the dental phenotype.

FAM20C is a Golgi casein kinase that phosphorylates secretory proteins such as FGF23 and SIBLING (Small Integrin-Binding Ligand, N-linked Glycoprotein) family [39, 40]. *Fam20c* is significantly expressed in mouse teeth and bone and transgenic mice studies have indicated a role in differentiation and mineralization of odontoblasts, ameloblasts, osteoblasts and osteocytes during tooth and bone development. FAM20C-deficient mice also have a prominent dental phenotype [41–43]. Ablation of the *Fam20c* gene in conditional knockout mice affects tooth and bone development by downregulation of SIBLING family of proteins such as DMP1 and DSPP and by increasing FGF23 in serum and promoting phosphate excretion and hypophosphatemia [44]. Our *FAM20C*-deficient dogs have a dental phenotype similar to mice and humans and provide a new research and preclinical model for this rare human bone disease. Unlike rodents, dogs have dental physiology similar to human, having both deciduous and permanent dentition.

In summary, we describe here the clinical and genetic characteristics of three new canine models for rare human bone disorders. While highlighting clinical and genetic similarities between canine and human conditions, our study have several implications; it indicates new physiological functions for the identified genes and provides new candidate genes to rare human diseases, establishes potential preclinical models, and finally enables the development of genetic tests for veterinary diagnostics and breeding purposes.

## Materials and Methods

### Ethics statement

Sample collection in Finland was ethically approved by the Animal Ethics Committee of State Provincial Office of Southern Finland (Finland, ESAVI/6054/04.10.03/ 2012). The collection of blood samples in Switzerland was approved by the “Cantonal Committee For Animal Experiments” (Canton of Bern; permit 23/10).

### Study cohorts

EDTA-blood and tissue samples were collected from privately owned dogs in Finland, US and Switzerland. The samples were stored at -20°C until genomic DNA was extracted using the semi-automated Chemagen extraction robot (PerkinElmer Chemagen Technologie GmbH). DNA concentration was determined either with the NanoDrop ND-1000 UV/Vis Spectrophotometer or Qubit 3.0 Fluorometer (Thermo Fisher Scientific Inc.). Pedigrees were drawn by the GenoPro genealogy software (http://www.genopro.com/), and utilizing the public dog registry by the Finnish Kennel Club (http://jalostus.kennelliitto.fi).

Clinical examinations for each condition are described in detail in the results section. CMO cases were diagnosed by radiography by local veterinarians. The developmental disorder in Wire Fox Terriers was investigated by radiography and CT. Neurological examination was performed for three and ophthalmoscopy for two of the affected dogs. A specialized dental veterinarian examined two of the affected Border Collies with dental hypomineralization, while the others were examined by local veterinarians. Clinical phenotype information was not available for dogs used as population controls.

### Genome-wide association study and fine mapping

Genome-wide association studies were performed in the CMO and VDEGS projects. For CMO, altogether 51 dogs including 10 affected and 41 control dogs, were genotyped using Illumina’s CanineSNP20 BeadChip of 22,362 validated SNPs. For VDEGS, a total of 15 dogs including 4 affected and 11 unaffected Wire Fox Terriers, were genotyped using Illumina’s HD array. The genotype data in both projects was filtered with a SNP call rate of >95%, array call rate of >95% and minor allele frequency of >5%. No individual dogs were removed for low genotyping and no SNPs were removed because of significant deviations from the Hardy-Weinberg equilibrium (p ≤ 0.0001). After frequency and genotyping pruning, 14,835 and 69,694 SNPs remained for analyses for CMO and VDEGS data, respectively. Basic case-control association test was performed by PLINK [45]. Genome-wide significance was ascertained with phenotype permutation testing (n = 10,000 for CMO and n = 100,000 for VDEGS). Fine mapping of the identified CMO locus was performed with 105 selected SNPs from a 1.9-Mb region (7,764,955–9,707,794 bp) on CFA5 (based on CanFam3.1). The SNPs were selected using Broad Institute SNP collection CanFam2.0. Genotyping was performed using the Sequenom (San Diego, CA, USA) iPLEX methodology at our local core facility in the FIMM Technology Centre, University of Helsinki, Finland. A total of 88 samples were genotyped including 8 cases and 8 controls in Cairn Terriers, 9 cases and 8 controls in Scottish Terriers and 29 cases and 26 controls in WHWTs. Association analysis was performed with PLINK using a single-marker association analysis.

### Targeted capture and resequencing

We performed a targeted sequence capture and next generation sequencing to identify the pathogenic variants. We used NimbleGen’s in-solution capture technology to enrich the target regions for sequencing (Roche NimbleGen, Madison, WI, USA). We captured 1.8-Mb region of CMO associated locus at position CFA5: 10,750,000–12,550,000 using two WHWT cases and controls with opposite haplotypes. The haplotypes were assessed manually using SNP genotype data. The same targeting experiment also contained samples from our other targeting projects including 8 Border Terriers, 12 Duck Tolling Retrievers, 8 Schipperkes and 4 Brazilian Terriers and these samples were used as additional controls. For target enrichment and sequencing of associated locus in Wire Fox Terriers, we captured a 3.3-Mb region at CFA26: 29,030,700–32,328,700 using two affected and two controls with opposite haplotypes and one obligate carrier. The filtered case-specific variants were further checked from 169 additional dogs in our variant database. Probes in the target regions were designed by Roche NimbleGen (Roche NimbleGen). Target enrichment, alignment and variant calling pipeline were performed as previously described [8]. Further data analysis was performed using open source R language and environment (http://www.r-project.org). Canine genome build CanFam3.1 was used as a reference sequence.

### Whole genome sequencing

The genetic causes of CMO in WHWTs and tooth attrition in Border Collies were studied by whole genome sequencing. In the CMO study, we performed whole genome sequencing of one affected WHWT dog and used 188 other available whole genomes as controls (**S2 Table**). A fragment library was prepared with a 290 bp insert size and collected a single lane of Illumina HiSeq2000 paired-end reads (2 x 100 bp). The reads were mapped to the dog reference genome using the Burrows-Wheeler Aligner (BWA) version 0.5.9-r16 with default settings. The Picard tools (http://sourceforge.net/projects/picard/) were used to sort the mapped reads by the sequence coordinates and to label the PCR duplicates. The Genome Analysis Tool Kit (GATK version v2.3–6) was used to perform local realignment and to produce a cleaned BAM file. Variant calls were then made using the unified genotyper module of GATK. Variant data was obtained in variant call format (version 4.0) as raw calls for all samples and sites flagged using the variant filtration module of GATK. Variant calls that failed to pass the following filters were labeled accordingly in the call set: (i) Hard to Validate MQ0 ≥ 4 & ((MQ0 / (1.0 * DP)) > 0.1); (ii) strand bias (low Quality scores) QUAL < 30.0 || (Quality by depth) QD < 5.0 || (homopolymer runs) HRun > 5 || (strand bias) SB > 0.00; (iii) SNP cluster window size 10. The SnpEff software together with the CanFam 3.1 annotation was used to predict the functional effects of detected variants. In addition to the SNP and short indel variant calling, large deletions contained in the candidate region were searched by visual inspection of the BAM file using the Integrative Genomics Viewer (IGV). In the Border Collie study, we whole genome sequenced altogether nineteen dogs, including three affected dogs, two carriers and fourteen unaffected Border Collies, in the Science for Life Laboratory in Stockholm, Sweden. The reads were processed using speedseq align module available in SpeedSeq suite to produce a duplicate-marked, sorted and indexed BAM file. The Genome Analysis Tool kit (version = 3.3.0-g37228af) was used to perform realignment around potential indel sites and base quality score recalibration using the known SNP variation available at the Broad Institute (https://www.broadinstitute.org/ftp/pub/vgb/dog/trackHub/canFam3/variation). Dual algorithms, Samtools mpileup (version samtools-1.2) and GATK haplotype caller module were used to detect variants and the variants from both algorithms were merged into variant call format (VCFv4.1). Annovar and SnpEff tools were used to annotate the variants to Ensembl, NCBI and Broad annotation databases to predict the functional effects of the variants. We identified on average ~6 million variants per sample and the sequencing coverage varied between 22-49x. The three affected dogs shared ~1.5 million homozygous variants. Filtering under recessive model against two carriers and fourteen controls left 2690 homozygous variants in total, of which five variants were in the predicted coding regions (1 indel, 2 non-synonymous, 1 synonymous).

### Genomic DNA analysis

Large numbers of dogs were genotyped for the identified variants using various protocols. Genotyping of individual dogs for the CMO variant was performed either by TaqMan assay (Applied Biosystems) or by sequencing a 786-bp PCR product using a forward primer (5-GGCTCCAGTCTAAGCCAGGT-3) and a reverse primer (5-AAGGAGTGCGCTCAAGACAG-3) flanking the SLC37A2 SNP. The PCR products were amplified with AmpliTaqGold360Mastermix (Life Technologies), and the products were directly sequenced using the PCR primers on an ABI 3730 capillary sequencer (Life Technologies) after treatment with exonuclease I (New England Biolabs) and rapid alkaline phosphatase (Roche). The sequence data were analyzed using Sequencher 5.1 (GeneCodes). Potential exonic splice enhancer (ESE) motifs were detected with ESEfinder 3.0 [46, 47].

Pathogenicity of the *FAM20C* c.899C>T variant was evaluated using web-based bioinformatic prediction tools SIFT [48] and PolyPhen-2 (genetics.bwh.harvard.edu/pph2) [49] was applied to evaluate the pathogenic effect of the mutation. SIFT score ranges from 0 to 1. The amino acid substitution is predicted to be damaging if the score is smaller than 0.05. PolyPhen-2 score ranges from 0 to 1. The amino acid substitution is predicted to be damaging if the score is bigger than 0.85.

Genotyping of *FAM20C* and *SCARF2* variants was performed by standard PCR with the following primers: *FAM20C*: 5-GCTTCTATGGCGAGTGTTCC-3 and 5-CCGGGATGTCTGAGTAAGGA-3; *SCARF2*:5-CAATCCCCGAGTGCTCTCC-3 and 5-AGGAAACTGCCCCCAAAGAG-3.

### Transcript analysis

Expression analyses were performed for the CMO and VDEGS projects. Blood samples were collected into PAXgene tubes (PreAnalytix) and RNA was isolated using PAXgene Blood RNA Kit (Qiagen). The cDNA synthesis was performed using SuperScriptIII enzyme (Invitrogen) with an oligo d(T)24V primer according to manufacturer’s instructions. To investigate possible aberrant splicing events, we amplified cDNAs from the junction of exons 7 and 8 to the junction of exons 17 and 18 of the *SLC37A2* gene (810 bp) using SequalPrep Long Polymerase (Invitrogen) with a forward primer GAATACCCAGAAGACGTGGAC and a reverse primer CCTCTGTCTCTGTTCAGGAATG in 16 WHWTs. *B2M* was used as a loading control. The identity of the amplicons was confirmed by Sanger sequencing. In the VDEGS project, the possible effect of the 2-bp deletion on the stability of the *SCARF2* transcript was investigated by RT-PCR. Total RNAs were isolated from skin samples from one affected and unaffected dog obtained in the postmortem autopsies. The cDNA synthesis was performed as described above. Forward 5-CAACCACGTCACTGGCAAGT-3 and reverse 5-TTACAGTGGGG CCCGTGG-3 primers were designed to amplify a 188-bp region between exon 6 and exon 8 of SLC37A2. Semi-quantitative analysis of the expression was determined and visualized by electrophoresis.

### Histological examination of teeth

Permanent and deciduous incisor and premolar teeth were removed for therapeutic reasons from two Border Collie bitches with clinically affected teeth. The dogs were from the same litter. Three teeth, two incisors and one premolar, were obtained from one dog. One incisor and the premolar were processed to ground sections and the other incisor to paraffin sections. From the other dog, four teeth, two incisors and two premolars, were obtained. One incisor and one premolar were processed to ground sections and the other incisor and the other premolar to paraffin sections. For comparison, deciduous teeth obtained from a healthy Border Collie were studied. One incisor was processed to ground sections and one tooth to paraffin sections. The teeth to be processed to ground sections (a procedure preserving the enamel with a proportionally high mineral content and sparse organic matrix) were fixed with 10% neutral buffered formalin, dehydrated and embedded in liquid methylmethacrylate monomer. After complete polymerization, started up by benzoylperoxide (2 g/l), the teeth were serially cut to 100–150 μm thick longitudinal sections with a rotating diamond-coated saw microtome, let dry and mounted unstained with DePex (Gurr, BDH, Poole, UK). For preparation to paraffin sections, the teeth were fixed with formalin, demineralized with EDTA (0.33 mol/l, pH 7.2), which leads to the loss of the enamel, and embedded in paraffin. A representative series of sections were longitudinally cut at 7 μm and stained with haematoxylin and eosin (HE).

## Supporting Information

S1 Fig The Border Collie pedigree with tooth wear. Dogs that were whole genome sequenced are indicated by open red box. Full segregation of the *FAM20C* c.899C>T variant with the disease according to recessive model is indicated.
(PDF)

S1 Table Summary of the targeted resequencing data in CMO. (DOCX)

S2 Table Summary of the breeds and the number of dogs used for variant filtering in the three studies. (XLSX)

S3 Table Summary of validation data of the *SLC37A2* c.1332C>T variant. (DOCX)

S4 Table Summary of the targeted resequencing data in a developmental syndrome in Wire Fox Terriers. (DOCX)

S5 Table Summary of validation data of the *SCARF2* c.865_866delTC variant in Wire Fox Terriers and other related breeds. (DOCX)

S6 Table Summary of validation data of the *FAM20C* c.899C>T variant in Border Collies and other breeds. (DOCX)
