# Supplementary material for: Molecular Characterization of Three Canine Models of Human Rare Bone Diseases: Caffey, van den Ende-Gupta, and Raine Syndromes
Source: PLoS Genet. 2016 May 17;12(5):e1006037. doi: 10.1371/journal.pgen.1006037 (PMC4871343; doi:10.1371/journal.pgen.1006037)

S1 Figure. The Border Collie pedigree with tooth wear. Dogs that were whole genome sequenced are indicated by open red box. Full segregation of the *FAM20C* c.899C>T variant with the disease according to recessive model is indicated.

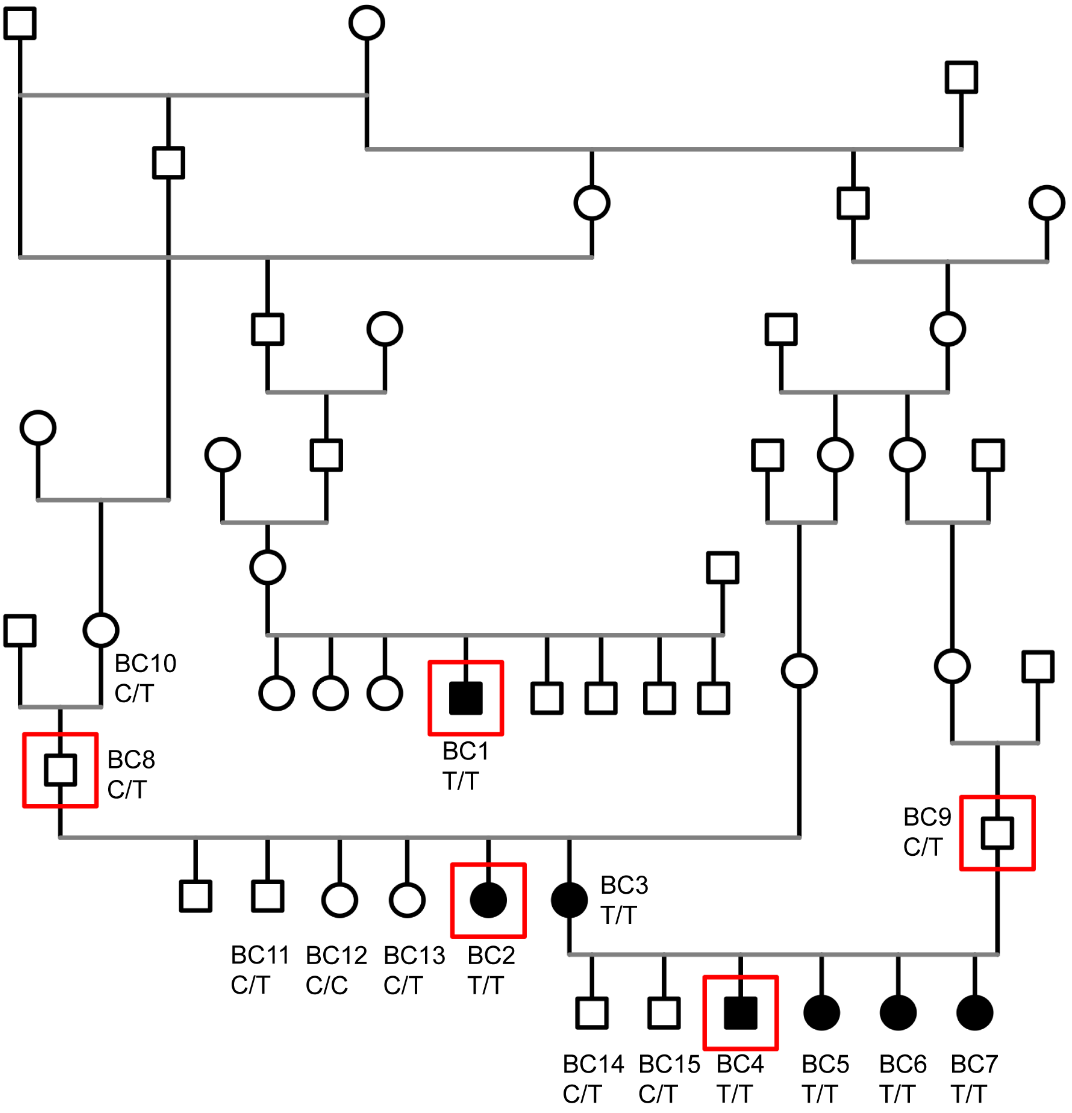

Supplement: S1 Fig — Dogs that were whole genome sequenced are indicated by open red box. Full segregation of the FAM20C c.899C>T variant with the disease according to recessive model is indicated. (PDF) [file pgen.1006037.s001.pdf]
