# Supplementary material for: Molecular Characterization of Three Canine Models of Human Rare Bone Diseases: Caffey, van den Ende-Gupta, and Raine Syndromes
Source: PLoS Genet. 2016 May 17;12(5):e1006037. doi: 10.1371/journal.pgen.1006037 (PMC4871343; doi:10.1371/journal.pgen.1006037)
Supplement: S1 Table — (DOCX) [file pgen.1006037.s002.docx]

**S1 Table**. Summary of the targeted resequencing data in CMO.

|  | **No. of reads** | **% mapped** | **Coverage** | **Total variants** | | **Exonic variants** | |
| --- | --- | --- | --- | --- | --- | --- | --- |
|  |  |  |  | **SNPs** | **Indels** | **SNPs** | **Indels** |
| WHWT case 1 | 29274625 | 99.14 | 49X | 5762 | 1923 | 50 | 3 |
| WHWT case 2 | 23014416 | 99.00 | 40X | 8240 | 2359 | 70 | 4 |
| WHWT control 1 | 24571490 | 99.09 | 40X | 6017 | 1948 | 54 | 4 |
| WHWT control 2 | 32389163 | 98.33 | 19X | 8055 | 1929 | 69 | 4 |
| Filtering according to recessive model* | | | | 1** | 2 | 1** | 0 |

*Novel homozygous variants shared with cases and absent in controls of WHWT and other breeds.

**Synonymous variant *SLC37A2* c.1332C>T
