# Supplementary material for: Molecular Characterization of Three Canine Models of Human Rare Bone Diseases: Caffey, van den Ende-Gupta, and Raine Syndromes
Source: PLoS Genet. 2016 May 17;12(5):e1006037. doi: 10.1371/journal.pgen.1006037 (PMC4871343; doi:10.1371/journal.pgen.1006037)
Supplement: S3 Table — (DOCX) [file pgen.1006037.s004.docx]

**S3 Table**. Summary of validation data for the *SLC37A2* mutation (c.1332C>T).

| **Dog breed** |  | **Total** | **wt, C/C** | **het, C/T** | **hom, T/T** |
| --- | --- | --- | --- | --- | --- |
| WHWT | CMO affected | 69 |  | 10 | 59 |
|  | Population control* | 626 | 297 | 265 | 64 |
|  | Total | 695 | 297 | 275 | 123 |
| Scottish terrier | CMO affected | 14 | 1 | 3 | 10 |
|  | Population control* | 235 | 195 | 40 |  |
|  | Total | 249 | 196 | 43 | 10 |
| Cairn terrier | CMO affected | 13 | 1 | 3 | 9 |
|  | Population control* | 95 | 83 | 12 |  |
|  | Total | 108 | 84 | 15 | 9 |
| 124 other breeds * | | 458 | 457 | 1** |  |
| Total (all breeds) | | 1385 | 909 | 334 | 142 |

*Clinically confirmed phenotype information in regard to CMO was not available for population controls and other breeds.

**Jack Russell Terrier
