# Supplementary material for: Molecular Characterization of Three Canine Models of Human Rare Bone Diseases: Caffey, van den Ende-Gupta, and Raine Syndromes
Source: PLoS Genet. 2016 May 17;12(5):e1006037. doi: 10.1371/journal.pgen.1006037 (PMC4871343; doi:10.1371/journal.pgen.1006037)
Supplement: S4 Table — (DOCX) [file pgen.1006037.s005.docx]

**S4 Table**. Summary of the targeted resequencing data in a developmental syndrome in Wire Fox Terriers (WFT).

|  | **No. of reads** | **% mapped** | **Coverage** | **Total variants** | | **Variants in ORF** | |
| --- | --- | --- | --- | --- | --- | --- | --- |
|  |  |  |  | **SNPs** | **Indels** | **SNPs** | **Indels** |
| WFT control 1 | 8,761,677 | 100 | 47.4 | 19619 | 2900 | 1018 | 171 |
| WFT control 2 | 6,878,828 | 100 | 37.2 | 19424 | 3095 | 1001 | 180 |
| WFT case 1 | 10,327,250 | 100 | 55.9 | 18038 | 2878 | 865 | 156 |
| WFT case 1 | 9,649,274 | 100 | 52.3 | 20956 | 3178 | 965 | 180 |
| Filtering according to recessive model | | | | 779 | 200 | 23 | 9 |
| No. of variants filtered against 169 exomes from other breeds* | | | | 28 | 9 | 0 | 1 |

*The breeds are listed in S2 Table
