# Supplementary material for: Molecular Characterization of Three Canine Models of Human Rare Bone Diseases: Caffey, van den Ende-Gupta, and Raine Syndromes
Source: PLoS Genet. 2016 May 17;12(5):e1006037. doi: 10.1371/journal.pgen.1006037 (PMC4871343; doi:10.1371/journal.pgen.1006037)
Supplement: S5 Table — (DOCX) [file pgen.1006037.s006.docx]

**S5 Table.** Summary of validation data of the *SCARF2* c.865_866delTC variant in Wire Fox Terriers and related breeds.

| **Breed** |  | **No. of dogs** | **wt, C/C** | **het, C/T** | **mut, T/T** |
| --- | --- | --- | --- | --- | --- |
| Wire Fox Terrier | Affected | 6 |  |  | 6 |
|  | Obligate carrier | 6 |  | 6 |  |
|  | Population control | 45 | 35 | 10 |  |
|  | Total | 57 | 35 | 16 | 6 |
| Brazilian Terrier |  | 28 | 28 |  |  |
| Jack Russell Terrier |  | 8 | 8 |  |  |
| Smooth Fox Terrier |  | 104 | 104 |  |  |
| Lakeland Terrier |  | 20 | 20 |  |  |
| Parson Russell Terrier |  | 10 | 10 |  |  |
| German Hunting Terrier |  | 10 | 10 |  |  |
| West Highland White Terrier |  | 28 | 28 |  |  |
| Welsh Terrier |  | 10 | 10 |  |  |
