# Supplementary material for: Molecular Characterization of Three Canine Models of Human Rare Bone Diseases: Caffey, van den Ende-Gupta, and Raine Syndromes
Source: PLoS Genet. 2016 May 17;12(5):e1006037. doi: 10.1371/journal.pgen.1006037 (PMC4871343; doi:10.1371/journal.pgen.1006037)
Supplement: S6 Table — (DOCX) [file pgen.1006037.s007.docx]

**S6 Table.** Summary of validation data of the FAM20C c.899C>T variant in Border Collies and other breeds.

| **Breed** | **Number of dogs** | **wt, C/C** | **het, C/T** | **mut, T/T** |
| --- | --- | --- | --- | --- |
| Border Collie, total | 191 | 162 | 22 | 7 |
| Affected | 7 |  |  | 7 |
| Obligate carrier | 2 |  | 2 |  |
| Population control | 182 | 162 | 20 |  |
| Akita | 7 | 7 |  |  |
| Alaskan Malamute | 8 | 8 |  |  |
| Australian Kelpie | 22 | 22 |  |  |
| Australian Shepherd | 16 | 16 |  |  |
| Bearded Collie | 8 | 8 |  |  |
| Belgian Shepherd, groenendael | 8 | 8 |  |  |
| Belgian Shepherd, tervueren | 8 | 8 |  |  |
| Collie, rough | 8 | 8 |  |  |
| Collie, smooth | 8 | 8 |  |  |
| English Springer Spaniel | 7 | 7 |  |  |
| German Shepherd | 8 | 8 |  |  |
| Great Dane | 7 | 7 |  |  |
| Icelandic Sheepdog | 8 | 8 |  |  |
| Lancashire Heeler | 8 | 8 |  |  |
| Miniature Schnauzer | 8 | 8 |  |  |
| Parson Russell Terrier | 8 | 8 |  |  |
| Saluki | 8 | 8 |  |  |
| Shetland Sheepdog | 15 | 15 |  |  |
| Spanish Water Dog | 8 | 8 |  |  |
| Swedish Vallhund | 8 | 8 |  |  |
| Other breeds, total | 186 | 186 |  |  |
